# Adhesion-related small bowel obstruction: deep learning for automatic transition-zone detection by CT

**DOI:** 10.1186/s13244-021-01150-y

**Published:** 2022-01-24

**Authors:** Quentin Vanderbecq, Roberto Ardon, Antoine De Reviers, Camille Ruppli, Axel Dallongeville, Isabelle Boulay-Coletta, Gaspard D’Assignies, Marc Zins

**Affiliations:** 1 Department of Medical Imaging, Saint Joseph Hospital, 185 rue Raymond Losserand, 75014 Paris, France; 2 Sorbonne Université, 75013 Paris, France; 3 Incepto-Medical, 128 rue La Boétie, Paris, France

**Keywords:** Abdomen, Small bowel, Obstruction, Neural networks, Tomography (X-ray computed)

## Abstract

**Background:**

To train a machine-learning model to locate the transition zone (TZ) of adhesion-related small bowel obstruction (SBO) on CT scans.

**Materials and methods:**

We used 562 CTs performed in 2005–2018 in 404 patients with adhesion-related SBO. Annotation of the TZs was performed by experienced radiologists and trained residents using bounding boxes. Preprocessing involved using a pretrained model to extract the abdominopelvic region. We modeled TZ localization as a binary classification problem by splitting the abdominopelvic region into 125 patches. We then trained a neural network model to classify each patch as containing or not containing a TZ. We coupled this with a trained probabilistic estimation of presence of a TZ in each patch. The models were first evaluated by computing the area under the receiver operating characteristics curve (AUROC). Then, to assess the clinical benefit, we measured the proportion of total abdominopelvic volume classified as containing a TZ for several different false-negative rates.

**Results:**

The probability of containing a TZ was highest for the hypogastric region (56.9%). The coupled classification network and probability mapping produced an AUROC of 0.93. For a 15% proportion of volume classified as containing TZs, the probability of highlighted patches containing a TZ was 92%.

**Conclusion:**

Modeling TZ localization by coupling convolutional neural network classification and probabilistic localization estimation shows the way to a possible automatic TZ detection, a complex radiological task with a major clinical impact.

**Supplementary Information:**

The online version contains supplementary material available at 10.1186/s13244-021-01150-y.

## Key points

- We combined CNN classification and probabilistic mapping to detect the transition zone of adhesion related SBO.
- Transition zones were most commonly located in the hypogastric region (56.9%).
- The coupled classification-network and probability-mapping model produced an area under the ROC curve of 0.93.

## Introduction

Small bowel obstruction (SBO) is a common nontraumatic surgical emergency, with approximately 400,000 admissions annually in the United States [1]. Among causes of SBO, the most common are adhesions [2].

All guidelines recommend computed tomography (CT) as the first-line imaging study for patients with suspected mechanical SBO [3, 4]. The goal is four-fold: (i) to confirm or refute the diagnosis of SBO and, when SBO is present, (ii) to locate the site of the obstruction, that is, the transition zone (TZ) (iii) to identify the cause, and (iv) to look for complications such as strangulation or perforation. Identifying the TZ or TZs (and determining their number) and establishing their locations is the first step in diagnosing the cause of SBO and differentiating the open-loop and closed-loop mechanisms [5]. A diagnosis of closed-loop SBO independently predicts ischemia [6] and help to decide whether surgery is needed in patients with adhesion-related SBO.

However, identifying the TZ or TZs is time-consuming and subject to inter-observer and intra-observer variability [7]. Automated TZ localization would therefore prove valuable for expediting the diagnosis in emergency cases and improving radiologists' performance in diagnosing the closed-loop mechanism.

The potential of machine learning to contribute to radiological diagnoses has expanded at a brisk pace in recent decades, initially thanks to increases in data-storage capabilities and subsequently due to the advent of parallel-processing hardware based on graphical processing units [8]. As a result, the number of studies of deep neural networks in medical imaging is escalating sharply. However, few teams are focusing on SBO. The only published classifications models were produced for standard abdominal radiographs [9–11]. No studies have used CT or 3D models, despite the recognized benefits of CT for diagnosing SBO and the probable contribution of 3D models, which may be comparable to that of multiplanar reformation [12].

The objective of this study was to build a 3D deep-learning model to help radiologists locate the TZ or TZs on CT scans from patients with adhesion-related SBO.

## Material and methods

### Dataset

All abdominopelvic CT scans performed at our institution between January 2005 and July 2018 were identified retrospectively. Of the 42,000 consecutive CT scan reports for the study period, 4098 contained the word or words “obstruction” and/or “intestine”. Of these 4098 CTs, 2472 had a health information system code for SBO. An abdominal expert radiologist determined that 1287 of the 2472 reports were for patients with mechanical SBO. Finally, 562 CTs from 404 patients with adhesion-related SBO were identified and annotated. Median patient age was 70 years (range 16–90 years) and there were 312 women and 250 men. Additional file 1: Table S1 describes the distribution of the study CTs and Additional file 1: Text T1 the CT acquisition technique.

**Fig. 1:**
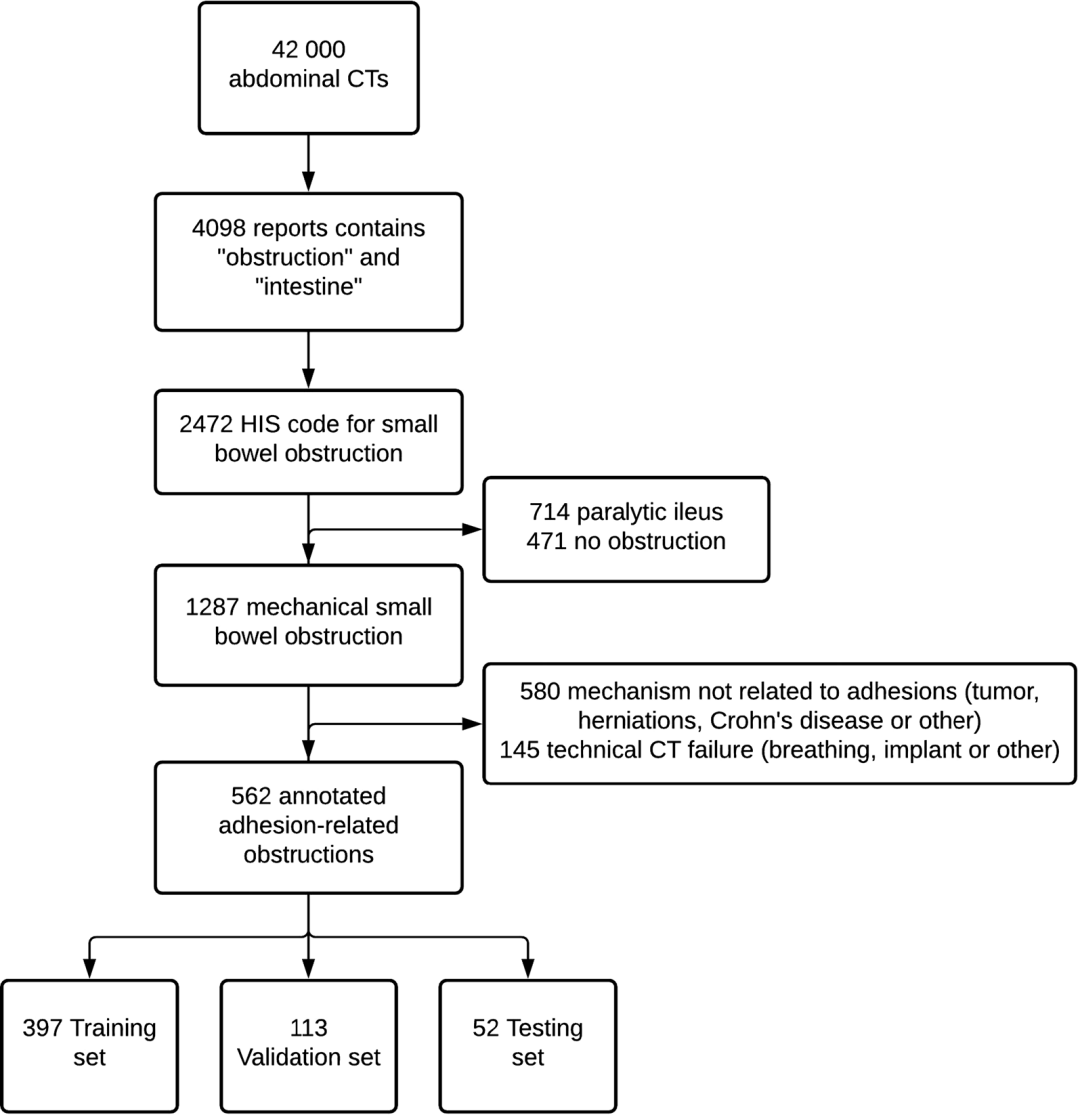
Flow chart of the data selection process. HIS: health information system

Of the 562 CTs, 247 were annotated by 7 radiologists with 2 to over 30 years of experience in abdominal CT. The remaining 315 CT scans were annotated by 9 residents who were previously trained in the process. All annotations were reviewed by study coordinator (Q.V.) and consensus has been reached with an expert with over 30 years of experience in abdominal radiology (M.Z.) for contentious cases between the study coordinator and radiologist annotator. These annotations were done using a platform built by Incepto-Medical (Paris, France). The annotators were asked to place one bounding box around a single TZ in case of open-loop obstruction and two bounding boxes for closed-loop obstruction [5]. Figure 1 summarizes the patient selection and reporting process.

Patients were randomly selected for training, validation, and testing, in proportions of 70%, 20%, and 10%, respectively. The split was performed at the patient level, with no overlap, i.e., volumes for a given patient appeared in only one of the three sets. Population parameters (age, sex) and annotator experience followed the same statistical distribution in all three sets (Additional file 1: Table S2).

### Preprocessing

CT scans provide high-resolution images. Volumes in the database were 3D arrays of size (nslices; 512; 512), where nslices was between 300 and 500. We applied the following preprocessing steps. First, a pretrained algorithm extracted the abdominopelvic region. Second, the random walk algorithm was used to segment the abdominopelvic region into a volume with a mean size of 300 × 400 × 300, with minimal void around the body.

This preprocessing method provided each of the abdominopelvic volumes with an anatomical bounding box. For a given volume selected as the reference, we used affine transformation to register all other abdominopelvic bounding box volumes. We summed the contributions of each box to generate a heatmap associated with the reference volume. This heatmap indicated the spatial distribution of TZs.

To locate each TZ, we used a patch-based approach to train a binary classification model. We performed three different experiments, splitting the abdominopelvic region into 27 (3 × 3 × 3), 64 (4 × 4 × 4), or 125 (5 × 5 × 5) patches. Each patch was classified in a binary manner, i.e., with or without a TZ, using the center of the annotated bounding box as the standard reference for TZ location. To train deep-learning classification models, each patch was resized to 64 × 64 × 64.

### Model and training parameters

**Fig. 2:**
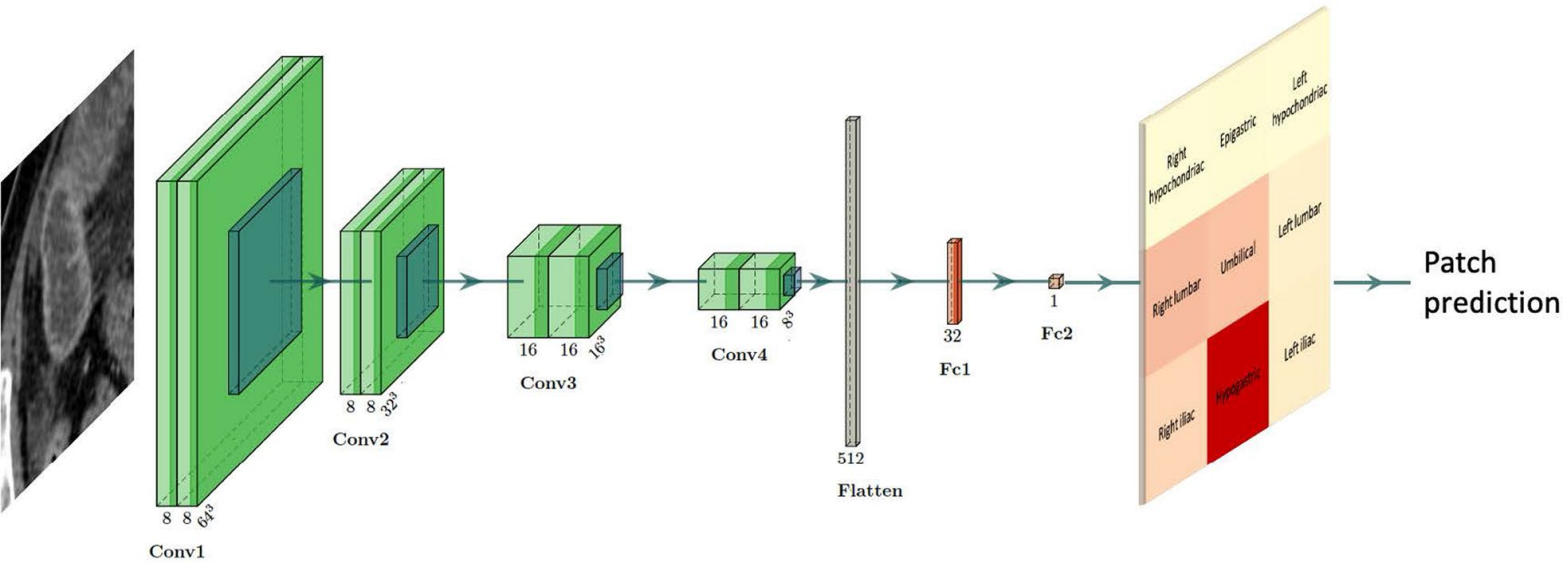
Model architecture. The 3D convolutional neural network has four convolution blocks followed by two dense layers. After the last layer, the classification score is multiplied by the spatial probability that the patch contains a transition zone, as computed on our training set

We modeled a convolutional neural network (CNN) with four convolution blocks followed by two dense layers (Fig. 2). Each convolution block was composed of two 3D convolution layers (kernel size 3) followed by Rectified Linear Unit (ReLU) activation and batch normalization then by a 3D MaxPooling layer (pool size 2). Each dense layer was followed by ReLU activation with a 0.5 dropout rate to prevent overfitting.

We used balanced cross entropy loss to increase the weights of positive sample classifications, thereby mitigating database imbalance. We also performed data augmentation on positive patches with random translation between 0 and 3 cm in each direction and random zoom between 0.8 and 1.2.

### Reduction of the search space for the transition zone (TZ)

The output of our model was a map overlaid on the abdominopelvic region. On the map, areas most likely to contain a TZ were highlighted. We used two methods to generate this map. The first highlighted patches if the CNN classification score was above the threshold maximizing the Youden index (sensitivity + specificity – 1, computed on the validation database) [13]. The second method highlighted patches based on the CNN classification score multiplied by the patch spatial probability of containing a TZ (or TZs), calculated on our training set.

### Classification performance

To compare classification models, we computed the area under the receiver operating curve (AUROC) and precision $\left( {\frac{True\,positive}{True\,postive + False\,positive}} \right)$.

### Search-space reduction versus risk of missing the transition zone (TZ)

To evaluate the practical benefits of our method, we compared the proportion of abdominopelvic volume that was highlighted (using both above-described methods) to the probability of missing a TZ. This last probability was computed on the test database.

## Results

### Transition zone (TZ) location

**Fig. 3:**
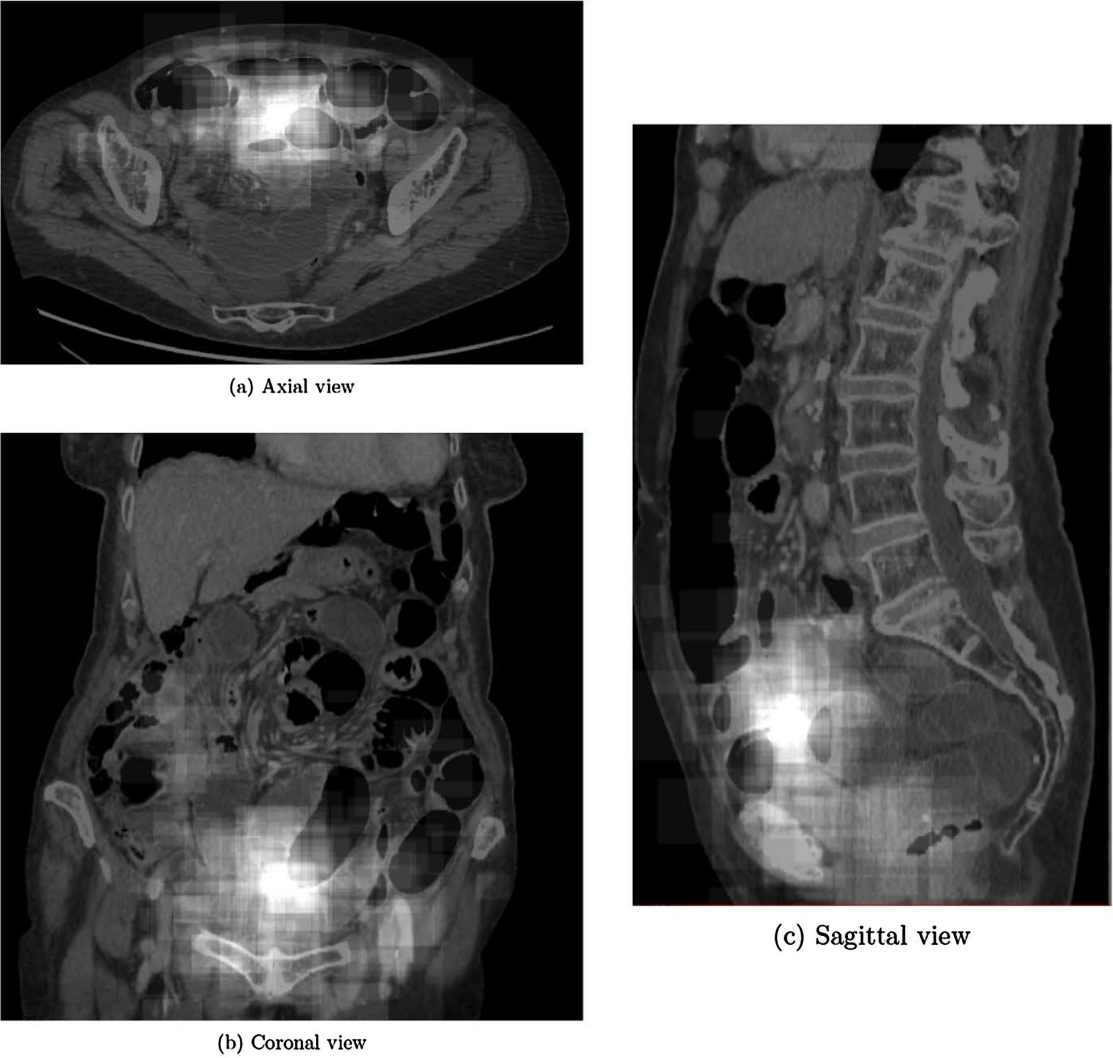
Heatmap of adhesion-related small bowel obstruction transition zone (TZ) localization. The images are shown with grey scale, with a maximum of 56.9% in white, to show the probability of presence of a TZ in any given reference volume

We first analyzed our dataset of 562 annotated CT scans. As outlined above, each bounding box was registered in a selected body, and a heatmap was built by summing the contributions of each box (Fig. 3). Analysis of the abdominal quadrant demonstrated that TZs predominated in the hypogastric quadrant (56.9%) and more than 85% of TZs are located in three regions (hypogastric, right lumbar and umbilical regions) (Table 1).

**Table 1:** Percentage of transition zones found in each of the six abdominal regions

Right hypochondriac	Epigastric	Left hypochondriac	Right lumbar	Umbilical	Left lumbar	Right iliac	Hypogastric	Left iliac
0	0.3	0.2	14.7	13.1	2.8	8.5	56.9	3.4

### Classification performance

**Fig. 4:**
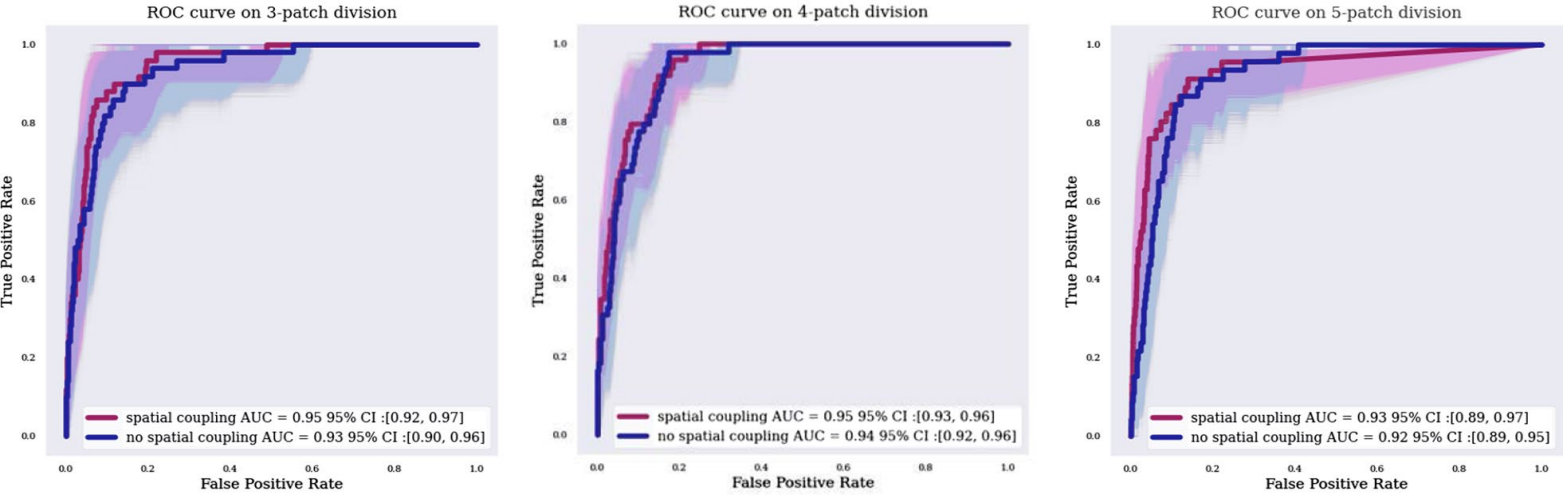
AUROC curves obtained with the different divisions of the abdomino-pelvic region. Divisions into 3 × 3 × 3 patches (3-patch division, left), 4 × 4 × 4 patches (4-patch division, center), and 5 × 5 × 5 patches (5-patch division, right)

We used the training set to train the CNN, the validation set to tune the optimization parameters (learning rate and stopping criteria of the training stage), and the test set for the final evaluation. We evaluated different abdomen partitions in 27 (3 × 3 × 3 partitions), 64 (4 × 4 × 4) and 125 (5 × 5 × 5) patches. And we achieve an AUROC of 0.95 (CI: 0.92–0.97), 0.95 (CI: 0.93, 0.96), 0.93 (CI: 0.89–0.97) (Fig. 4) and a mean precision of 0.43 (CI: 0.30–0.57), 0.31 (CI: 0.19–0.45) and 0.13 (CI: 0.08–0.22), respectively. The differences in these results are attributable to the dataset imbalance: because the prevalence of TZs across all patches of each patient was very low, both good sensitivity and good specificity were achieved but mean precision was very low (Additional file 1: Table S3).

**Fig. 5:**
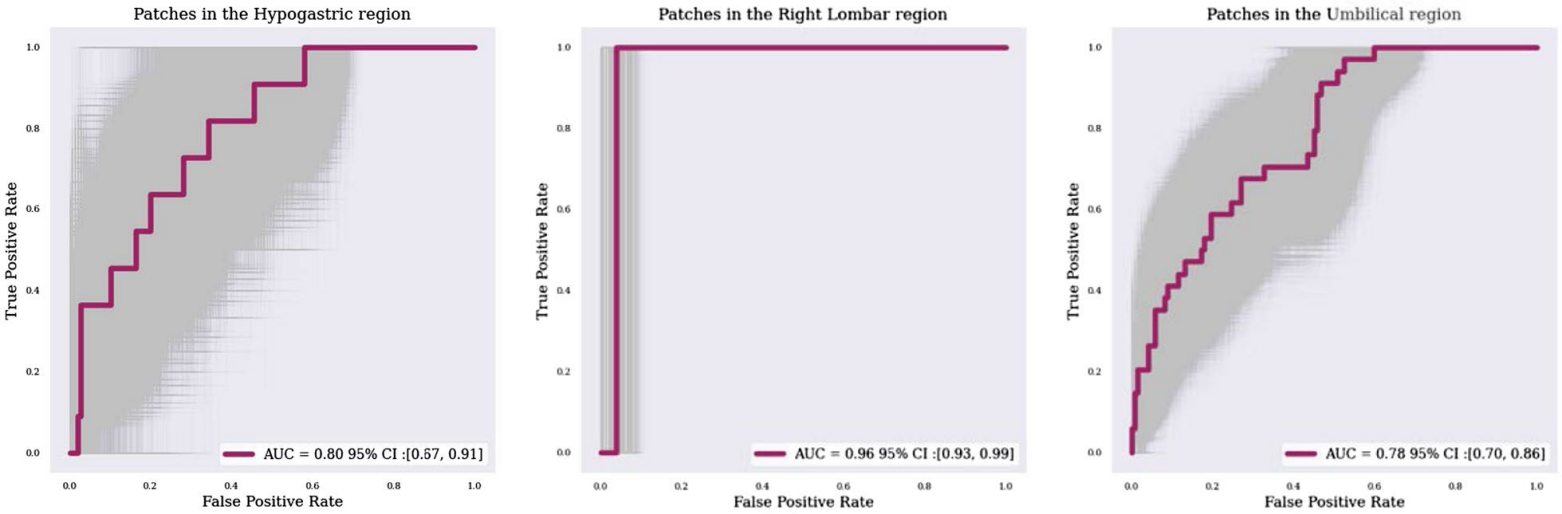
AUROC curves in three specific abdominal regions: hypogastric (left), right lumbar (center), and umbilical (right). These curves were computed by summed the patch classification scores in hypogastric, lumbar and umbilical regions

Moreover, the AUROC values for patches positioned on specific anatomical regions varied widely (Fig. 5). For instance, the value was very high for the right lumbar region but was only 0.80 in the umbilical and hypogastric regions.

### Search-space reduction versus risk of missing the transition zone (TZ)

To integrate this spatial heterogeneity, each patch classification score was weighted by its spatial probability of containing a TZ (Table 1).

The AUROC for our best weighted patch classification model (125 patches) was 0.93. While this value may be considered acceptable, it was insufficient for TZ localization. To locate the TZ, 125 predictions were required, and an acceptable patch classifier would find a single positive patch. In our case, the false-positive rate (maximizing the Youden index) was 0.14.

Under the hypothesis of a single patch containing the TZ, the number of highlighted patches that were erroneously classified followed the binomial law of parameters (124, 0.14). The mean number of erroneously classified patches was 17/124. We thus chose to exploit our classifier as a highlighting map identifying the abdominopelvic regions most likely to contain the TZ.

**Fig. 6:**
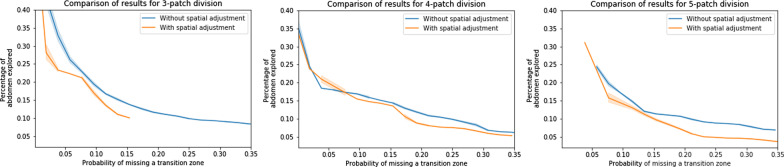
Model abdominopelvic-volume output according to the false-negative ratio, with or without spatial adjustment

With spatial weighting, the proportion of highlighted abdominopelvic volume decreased while keeping an acceptable probability of missing the TZ (Fig. 6).

Figures 4 and 6 shows the impact of this spatial adjustment on the percentage of abdominopelvic volume highlighted by the model. The AUROC value improved only by 0.01 or 0.02, depending on patch division (Fig. 5), with no change in the false-negative rate, but the searched abdominopelvic volume decreased by 4% (Fig. 6).

Our best map (125 patches with spatial coupling) highlighted the TZ with a probability of 93% while covering only 15% of the abdominopelvic volume. Additional file 1: Figure S1 shows an example of an output result.

## Discussion

We developed a machine-learning model to reduce the TZ search space on abdominopelvic CT scans of patients with suspected SBO. To that end, we built a large annotated SBO database.

Several factors may explain why only two earlier studies have evaluated an artificial-intelligence approach to SBO diagnosis, despite the high frequency of mechanical SBO among nontraumatic surgical emergencies [9, 10]. It should be noted that these studies focused on standard radiographs, which are no longer recommended for diagnosing SBO [4]. First, developing an artificial-intelligence model for TZ detection requires the establishment of a large database by highly experienced radiologists. Second, the abdominopelvic volume in which the TZ may reside is large compared to other medical volumes such as the intracranial space. Third, the abdominopelvic volume varies considerably across individuals due to differences in the amount of intra-abdominal fat and in intestinal motion. Finally, TZ detection is facilitated by a 3D assessment [12], which requires a more complex model than those used in radiography.

To precisely locate a transition zone, high resolution imaging is needed. Training a model using the whole CT volume at such resolution requires high performance GPUs. These GPUs are incompatible with routine clinical use because of their computational costs. To be able to train at high resolution we chose a balance between performance objective and technical limits and performed a patch extraction before classification as other detection networks do [14].

Locating the TZ is not only of diagnostic value but may also help to plan surgical procedures, for instance trocar placement for laparoscopy. In a recent metanalysis, CT was 92% sensitive and 87% specific for detecting the TZ [15]. However, this good performance comes at the cost of spending considerable radiologist time. TZ location is the first step toward establishing the cause of SBO and evaluating the risk of ischemia. Unfortunately, radiologist precision for detecting a closed-loop mechanism is poor. In a study of 88 patients who had surgery for SBO, including 24 with a closed-loop mechanism, sensitivity of CT interpreted by radiologists for detecting this mechanism was only 53% (95% confidence interval, 44%–63%) and specificity was 83% (79%–87%) [7]. Furthermore, inter-observer agreement for closed loop detection was poor to moderate (*k* = 0.39–0.63).

To facilitate TZ detection on CTs, we built a model designed to decrease the search volume in the abdominopelvic region, accelerate the emergency-CT workflow [16, 17], and increase the appropriateness of treatment decisions. We expected TZs to predominate in the right iliac region, since a leading cause of adhesion-related SBO is prior abdominal surgery and appendectomy is a common procedure [18]. Unexpectedly, TZs predominated significantly in the hypogastric region. We are aware of a single study of TZ location, which was determined only in the sagittal plane [19]. The results showed that the closed-loop mechanism was most common when there was more than one TZ and the TZs were located posteriorly, within 7 cm anterior to the anterior edge of the spine. The predominance of TZ in hypogastric region may be related to the high frequency of gynecological surgical procedures. Furthermore, the appendix is sometimes located in the pelvis.

Coupling a deep-learning classification model with a priori spatial knowledge improved model performance in our study. Although this is a simple logical concept in medicine, few deep-learning models applied to the medical domain leverage it. Only a few recent models in the field of natural images have started to use spatial probability estimation [20].

A limitation of our study relates to external validity, since the data came from a single center and all the models of CT machines used were from the same manufacturer. This limitation applies chiefly to our testing set. Since the accuracy for identifying a patch containing a single TZ is limited, our model is not optimal for clinical practice use. Much work remains to be done to build machine-learning models that approach the performance of an abdominal imaging radiologist. We only studied adhesion-related SBO as they represent more than 60% of SBO etiologies [21]. A further study should include all etiologies. The data ground truth may be open to criticism: we relied on annotations made by specialists on CT scans as opposed to a biological reference standard such as those often available in other fields (e.g., oncology). Radiologists have demonstrated good sensitivity and specificity for TZ localization [15], despite the significant heterogeneity in this parameter. The best reference standard for TZ location by CT remains unclear. In particular we can assume that a surgical reference, could be optimal. However, an important proportion of adhesive-related SBO are managed non operatively, and to obtain a representative population of bowel obstructions we cannot restrict our inclusions to operated SBO. Moreover, the possibility of precisely locating the junctional zone in space during surgery is subject to discussion.

Finally, to build a prediction model for the management of adhesion related SBO, the first step is to locate the TZ. This information helps to determine whether the mechanism is open or closed loop the latter being part of the risk factors for intestinal ischemia [19]. We now plan to develop models that predict ischemia, with annotation on CT scans of decreased bowel-wall enhancement and diffuse mesenteric haziness.

To conclude, our study makes two important contributions. First, the results show that the hypogastric region is the most common location of TZs in patients with adhesion related SBO. Second, our model combining CNN with a probabilistic mapping reduced the abdominopelvic TZ-search volume. This is a first step towards the development of an effective machine learning system that will save valuable time when performing complex and often urgent TZ identification and localization.

## Supplementary Information

**Additional file 1.** **Table S1:** Distribution of the CT machines used. **Table S2:** Distribution of patient demographics and experience of annotator. **Table S3:** AUROC and precision results on the test set. **Supplementary Text S1:** Additional technical information. **Figure S1:** Example of result.

## Data Availability

The scan datasets generated during the current study are not publicly available due to the fact that the patients who authorized the use of the data for this study were not questioned about the possibility of making these same data public.

## Abbreviations

AUROC: Area under the receiver operating characteristics curve
CNN: Convolutional neural network
CT: Computed tomography
SBO: Small bowel obstruction
TZ: Transition zone
