# Supplementary material for: Adhesion-related small bowel obstruction: deep learning for automatic transition-zone detection by CT
Source: Insights Imaging. 2022 Jan 24;13:13. doi: 10.1186/s13244-021-01150-y (PMC8787000; doi:10.1186/s13244-021-01150-y)
Supplement: Supplementary file 1 — Additional file 1. Table S1: Distribution of the CT machines used. Table S2: Distribution of patient demographics and experience of annotator. Table S3: AUROC and precision results on the test set. Supplementary Text S1: Additional technical information. Figure S1: Example of result. [file 13244_2021_1150_MOESM1_ESM.docx]

**ELECTRONIC SUPPLEMENTARY MATERIAL**

**Adhesion-Related Small Bowel Obstruction: Deep Learning for Automatic Transition-Zone Detection by CT**

Table of contents

Supplementary Tables 2

Supplementary Table S1: Distribution of the CT machines used 2

Supplementary Table S2: Distribution of patient demographics and experience of annotators 2

Supplementary Table S3: AUROC and precision results on the test set 2

Supplementary text 3

Supplementary Text S1 3

Supplementary Figure 4

Supplementary figure S1: Example of result 4

# Supplementary Tables

## **Supplementary Table S1: Distribution of the CT machines used**

| CT model | Training Set | Validation Set | Test Set |
| --- | --- | --- | --- |
| LightSpeed VCT | 48.9 | 59.3 | 50.0 |
| Optima CT660 | 4.0 | 3.5 | 1.9 |
| Revolution CT | 1.5 | 0.9 | 0.0 |
| Revolution EVO | 39.3 | 30.1 | 36.5 |
| Revolution Frontier | 6.3 | 6.2 | 11.5 |

The data are the percentages of image volumes acquired with each scanner model within each of the three sets.

## **Supplementary Table S2: Distribution of patient demographics and experience of annotators**

|  | Training Set | Validation Set | Test Set |
| --- | --- | --- | --- |
| Median age, years | 72 | 73 | 69 |
| Sex | 222 F / 175 M | 60 F / 53 M | 30 F / 20 M |
| Experienced Annotator | 43 % | 44 % | 50 % |

## **Supplementary Table S3: AUROC and precision results on the test set**

|  | 3 patches | | 4 patches | | 5 patches | |
| --- | --- | --- | --- | --- | --- | --- |
|  | Without spatial adjustment | With spatial adjustment | Without spatial adjustment | With spatial adjustment | Without spatial adjustment | With spatial adjustment |
| AUROC | 0.93 | 0.95 | 0.94 | 0.95 | 0.92 | 0.93 |
| Precision | 0.39 | 0.43 | 0.24 | 0.31 | 0.07 | 0.13 |

AUROC: area under the receiver operating characteristics curve

# Supplementary text

## **Supplementary Text S1**

All CT images were obtained using a 64-detector machine (described in Supplementary Table S1), with 1.375 pitch, 0.7 tube rotation time, and 120 kV. Images were first acquired without contrast material and with a nominal section thickness of 0.625 mm, reconstruction section thickness of 1.25 mm, and 1.25-mm gap. Then, an iodinated contrast agent (iopromide, iohexor, or iomeprol) was administered intravenously in a dose of 1.5 mL/kg and at a flow rate of 3 mL/s. Images were acquired at the portal phase (70 s after the injection), with a section thickness of 0.625 mm, reconstruction section thickness of 1.25 mm, and 1.25-mm gap. Oral contrast material was not used in any of the patients.

# Supplementary Figure

## **Supplementary figure S1: Example of result**


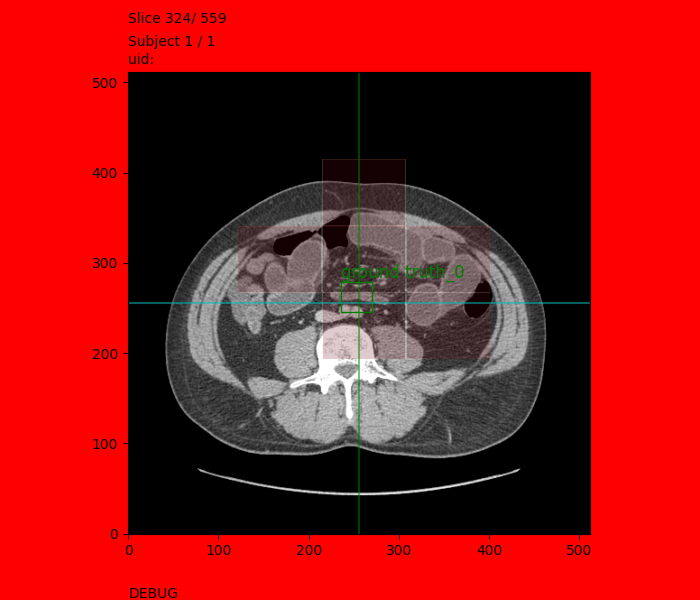

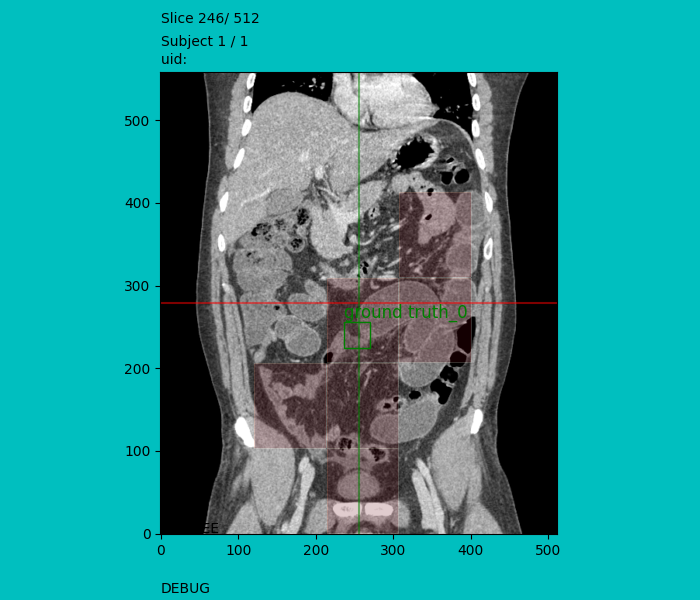


a

b

1. Axial view centered on transition zone point. b. Coronal view centered on transition zone point.
